# Prevalence and risk factors of atopic dermatitis in Chinese children aged 1–7 years: a systematic review and meta analysis

**DOI:** 10.3389/fpubh.2024.1404721

**Published:** 2024-07-31

**Authors:** Xueting Ma, Zhiyin Xie, Yu Zhou, Hui Shi

**Affiliations:** Department of Dermatology, Changping District Hospital of Traditional Chinese Medicine, Beijing, China

**Keywords:** children, atopic dermatitis, prevalence, meta-analysis, systematic review

## Abstract

**Introduction:**

Atopic dermatitis (AD) is a common, chronic, recurrent inflammatory skin disease. To date, no meta-analysis have been conducted on the prevalence and risk factors of AD in children aged 1–7 years in Mainland China.

**Methods:**

We conducted a meta-analysis of the prevalence and risk factors of AD among children aged 1–7 years in China. Chinese and English publications were searched in Chinese and English databases on AD epidemiology published between 1999 and 2023. Two researchers independently screened the literature, extracted the data, and evaluated their quality. A meta-analysis was performed using a random-effects model (I^2^ > 50%) with 95% confidence intervals (CIs) for the forest plots. Data were processed using the RevMan 5.3.

**Results:**

Nineteen studies (data from 127,660 samples) met the inclusion criteria. The pooled prevalence of AD in Chinese children aged 1–7 years was 8%. Over the last decade, the prevalence of AD has increased. The prevalence of AD among children in southern China was higher than that in northern China and was the highest at the provincial level in Zhejiang, Shanxi, and Anhui. The prevalence of AD was dependent on the family history of allergy, passive smoking, households with pets, plush toys, and residential area.

**Discussion:**

The prevalence of AD in children (age 1–7 years) in China has increased. Further studies are needed to monitor the prevalence of AD in Chinese children. Therefore, early prevention and screening should be performed for children with a family history of AD, and their living environment should be improved to reduce allergen stimulation, thus reducing the development of AD.

## 1 Introduction

Atopic dermatitis (AD) is a common inflammatory skin disease characterized by recurrent itching in children (1). The typical clinical features of AD are desiccation, erythema, papules, and exudation (2). Skin damage and itching affect the body, mood, and sleep of children and reduce their quality of life (3). In addition, AD increases a child's risk of attention deficit hyperactivity disorder (ADHD) and autism spectrum disorder (ASD) (4). Many patients with AD develop asthma and/or allergic rhinitis later in life (atopic march); asthma can lead to serious morbidity and even mortality (1). The pathogenesis of AD is complex. Genes, epidermal dysfunction, immune disorders, lifestyle, climate, air pollution, decreased diversity of the skin and intestinal microbiota, and psychological factors are believed to play roles in the pathogenesis of AD (5). Worldwide, approximately 15%−30% of children are affected by AD, a common chronic skin disease (6). AD places pressure and burden on children and their family members in terms of economic, mental, and social activities, and insomnia caused by itching is an important problem for children and family members (7).

The prevalence of AD increased in parts of Africa, East Asia, Western Europe, and Northern Europe from 1990–2010 (8). In addition, a high prevalence of AD has been reported in Sweden, Japan, New Zealand, the United Kingdom, Portugal, and Thailand (9–11). Studies have shown that the prevalence of AD in China is also on the rise. In 2004, 49,241 children from 10 provinces and cities were investigated, showing a prevalence of 2.8% (12). A subsequent study conducted in 2016 among 13,989 children from 12 provinces and cities in China reported a prevalence of 12.9% (13). However, with the rapid development of the Chinese economy and the acceleration of urbanization, the prevalence of AD in children may continue to rise. This may be caused by the interaction of genetic and environmental factors, but the detailed cause and pathogenesis are not clear. Therefore, continuous epidemiological investigation of AD in China is important.

Epidemiology is the discipline that studies the distribution and influencing factors of diseases and health conditions in a specific population to prevent, control, and eliminate diseases and promote health (14). Epidemiological investigations play an important role in the study of epidemic factors, etiology, natural history, and prevention and control of diseases (15). China has a vast territory and owing to the influence of the natural environment, there are great differences in the production, lifestyles, cultural customs, and other aspects of people in different regions, which have a certain impact on the distribution of AD. The prevalence of AD in children varies by region (12). The prevalence rate in urban children is higher than that in rural children (16). However, these are only summaries or cross-sectional surveys of the prevalence and risk factors; systematic reviews and meta-analyses of regional differences and risk factors for AD prevalence in Chinese children are scarce. Therefore, this study conducted a meta-analysis of the prevalence of AD in children in China and a systematic review of the collected research data to elucidate the total prevalence of AD in children in China and its risk factors. This study aimed to elucidate the epidemiological characteristics of AD in Chinese children and to provide supplementary data for a global epidemiological study of AD.

## 2 Materials and methods

### 2.1 Study search

This systematic review was conducted in accordance with the methods and recommendations of the PRISMA Extension Statement for Reporting Systematic reviews (17). We identified studies published in English between 1999 and 2023. English language databases (PubMed, Google Scholar, Cochrane Library, and Clinical Trials) and Chinese language database (CNKI, Cqvip, WANFANG data, and Baidu scholar) were searched using “child,” “children,” “Atopic Dermatitis,” “eczema,” “prevalence,” “incidence,” “epidemiology,” “epidemiologic,” “incidence,” “risk factors,” or variants and combinations of these keywords. Take “China” or “Chinese,” as crowd qualifiers. The references included in the studies were reviewed to identify all potentially eligible studies.

### 2.2 Inclusion and exclusion criteria

The following studies were included in this systematic review and meta-analysis: (1) the publications collected in this study were cross-sectional studies on the prevalence and epidemiology of AD published between 1999 and 2023; (2) the study samples for these publications were age-specific and included children aged 1–7 years; (3) the studies included in these publications were conducted on mainland Chinese populations; (4) identified the prevalence of AD or provided data that can be used to calculate the prevalence; and (5) all children in the study were diagnosed with AD using clear diagnostic criteria. Additionally, the following studies were excluded: (1) non-research-based publications, such as reviews, press releases, newsletters, and forums; (2) incomplete and valid data could not be extracted; (3) sample time, sample size, and prevalence not specified in the study; (4) unclear diagnostic criteria or unconventional diagnostic tools; (5) the sample age for the study did not include children aged 1–7 years; and (6) study population size of fewer than 30 cases.

### 2.3 Data extraction and quality assessment

The selected publications were independently evaluated by two reviewers according to established inclusion criteria. First, the titles and abstracts were quickly reviewed to eliminate irrelevant studies. The full texts of the remaining studies were then evaluated and screened for readability. If there was a disagreement, the study was independently evaluated by a third reviewer until a consensus was reached on the inclusion criteria. The quality of the selected publications was estimated using the Newcastle-Ottawa Scale (NOS). The full score is 10 points; studies with ≥5 points can be included in the meta-analysis (18). The included studies were assessed in a single-blinded manner. Data extraction tables were created using Microsoft Office Excel. Information extracted from the included studies included total sample size, number of patients, geographic location, sex, urban and rural area, author, and year of publication.

### 2.4 Data analysis and synthesis

A random-effects model was used for the eligible studies. The meta-analysis was performed using Review Manager 5.3 (Copenhagen: The Nordic Cochrane Centre, The Cochrane Collaboration, 2014). Forest plots were used to summarize the estimates with 95% confidence intervals (CIs). The heterogeneity index among the included studies was determined using Cochran's Q test (chi-square test) and Higgins' *I*^2^ statistics. If the heterogeneity test showed *P* ≥ 0.1 and *I*^2^ ≤ 50%, this indicates homogeneity between studies, and the fixed-effects model can be used for combined analysis. *P* < 0.1 and *I*^2^ > 50% indicated heterogeneity between studies. Sensitivity analysis or subgroup analysis was then used to determine the source of heterogeneity. If the *I*^2^ statistic was significant (*I*^2^ > 50%), a random effects model was used; otherwise, a fixed effects model was used. Statistical significance was set at *P* < 0.05, and a 95% CI was reported.

## 3 Results

### 3.1 Selection and description of included studies

**Figure 1:**
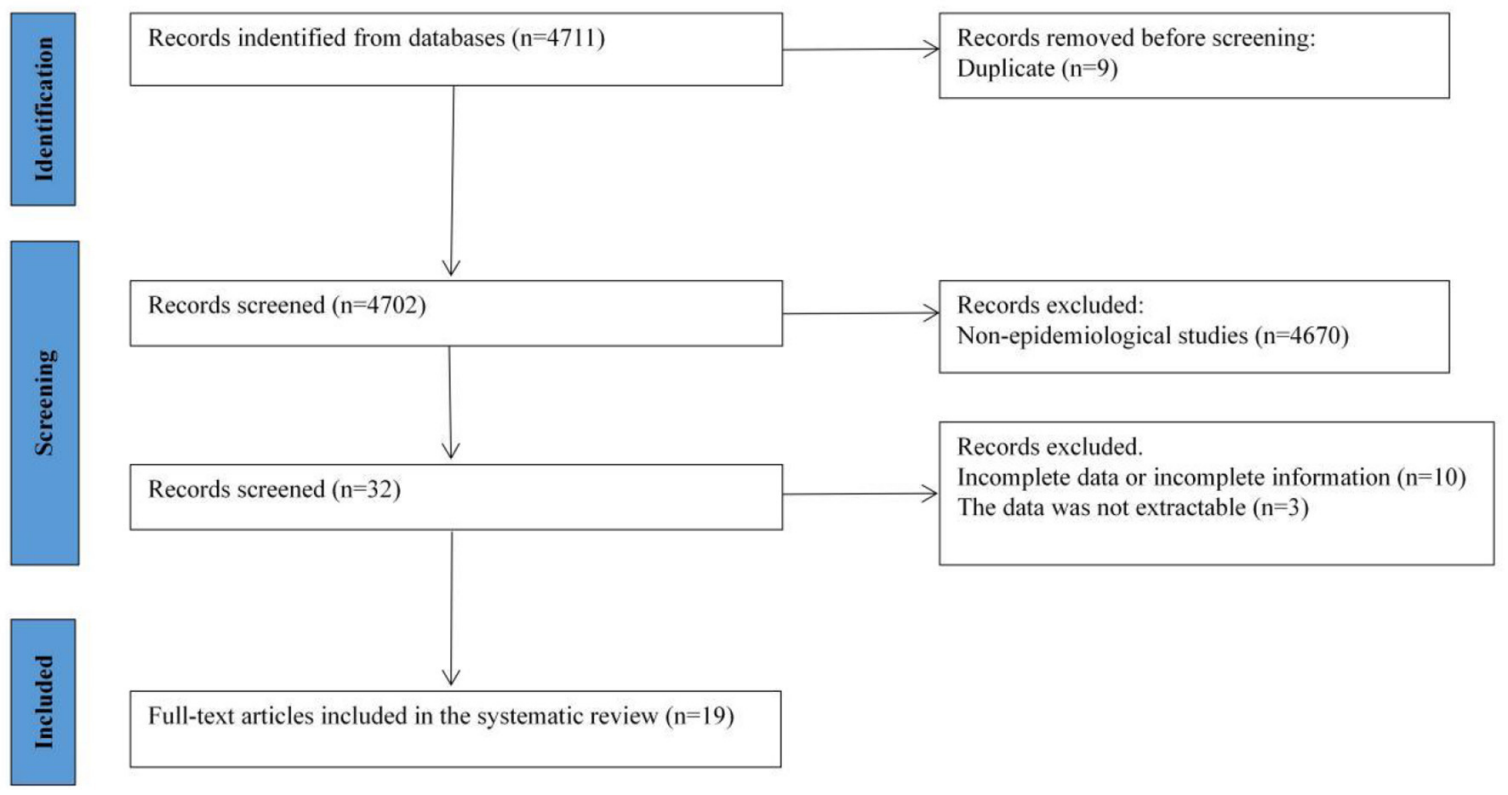
PRISMA flow diagram of selection of studies.

A total of 4,711 Chinese and English publications were identified using the database search strategy. After reviewing titles and abstracts, 32 publications were considered for full-text evaluation. After eliminating 13 publications with incomplete data or data that could not be extracted, 19 were included in the meta-analysis and data extraction (Figure 1). All included studies were single cross-sectional studies conducted from 1999–2023 with sample sizes ranging from 240–49,241. According to our criteria, all included publications with ≥5 points and could be included in the meta-analysis. A cross-sectional study was conducted for all publications and the prevalence of AD was calculated (Table 1).

**Table 1:** Collected publications on AD in Chinese children.

**References**	**Sampling year**	**No. examined**	**No. positive**	**Prevalence, %**	**Study design**
Xiao et al. (19)	2021	2,026	97	4.8	Cross-sectional
Wang et al. (20)	2020	3,489	209	6.0	Cross-sectional
Wei et al. (21)	2012	7,872	212	2.7	Cross-sectional
Xu et al. (16)	2010	10,436	870	8.3	Cross-sectional
Xu et al. (22)	2012	4,784	526	11.0	Cross-sectional
Gu et al. (23)	1999	2,249	20	0.9	Cross-sectional
Gu et al. (12)	2004	49,241	1,371	2.8	Cross-sectional
Zhou et al. (24)	2020	1,118	9	0.8	Cross-sectional
Zhou (25)	2017	418	29	6.9	Cross-sectional
Wang et al. (26)	2007-2008	620	4	0.6	Cross-sectional
Cai et al. (27)	2010	3,766	578	15.3	Cross-sectional
Liu et al. (28)	2019	5,900	150	2.5	Cross-sectional
Zeng et al. (29)	2002	3,708	108	2.9	Cross-sectional
Yan et al. (30)	2011	2,457	291	11.8	Cross-sectional
Zong et al. (31)	2014	13,061	738	5.7	Cross-sectional
Ji et al. (32)	2017	782	212	27.1	Cross-sectional
Guo et al. (13)	2016	13,989	1,811	12.9	Cross-sectional
Ji et al. (33)	2020-2021	240	57	23.8	Cross-sectional
Zhang (34)	2017	1,504	178	11.8	Cross-sectional

### 3.2 Prevalence of childhood AD

**Figure 2:**
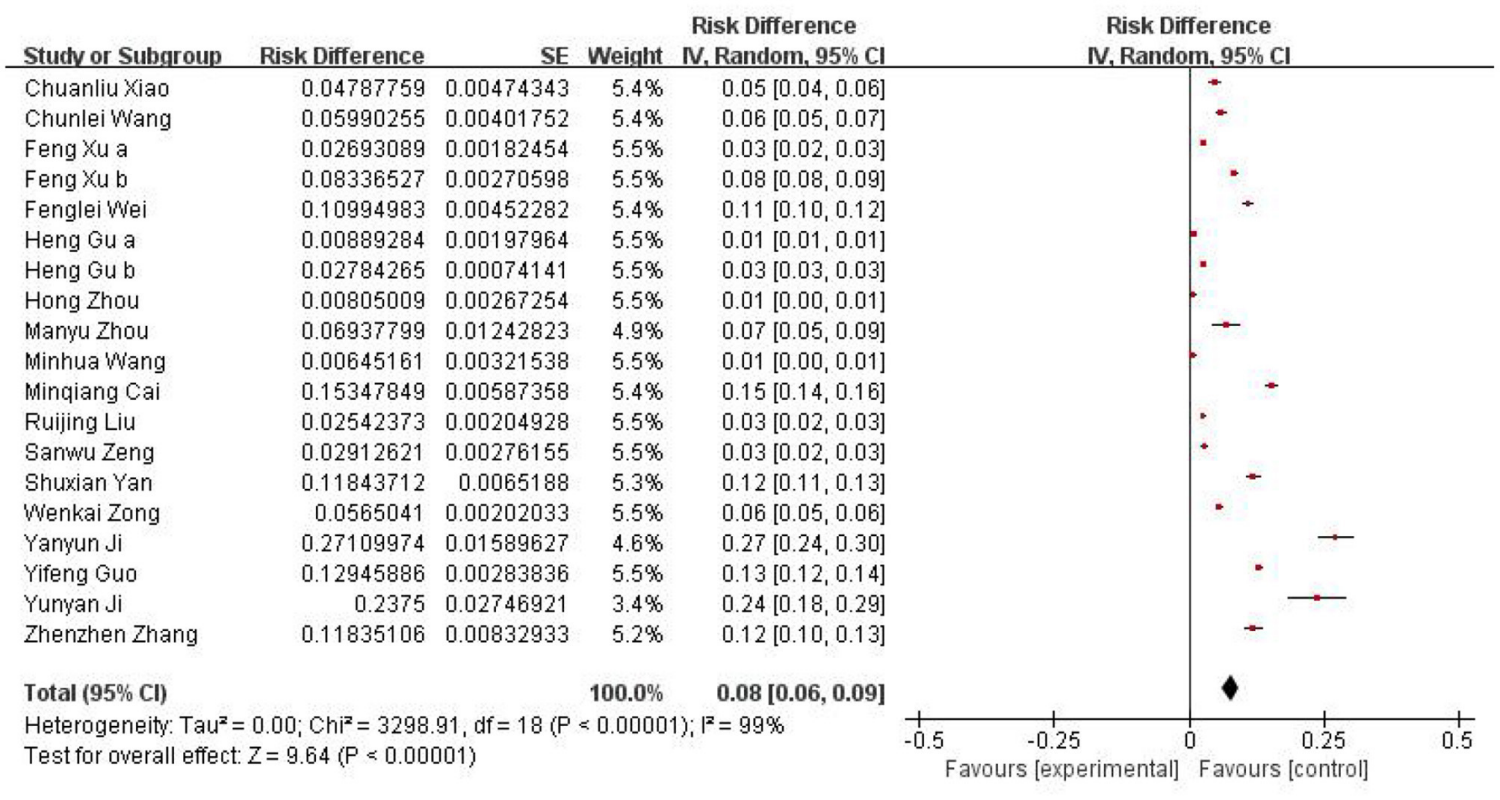
A forest plot for the current prevalence of atopic dermatitis (AD) in children of China.

**Figure 3:**
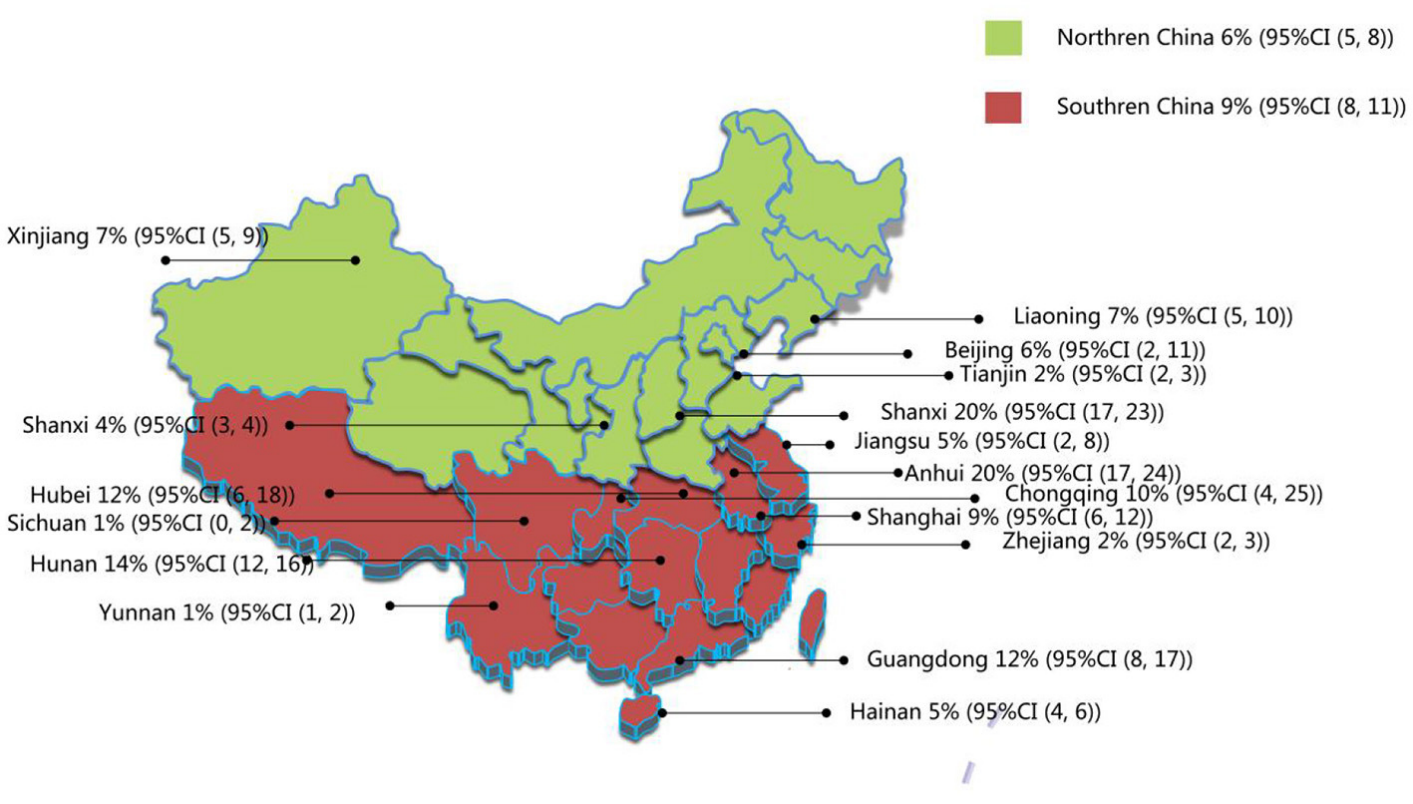
Regional distribution of current prevalence of AD in Chinese children. Northern China includes Liaoning, Jilin, Heilongjiang, Beijing, Tianjin, Hebei, Shanxi, Inner Mongolia, Henan, Shanxi, Shandong, Gansu, Qinghai, Ningxia, and Xinjiang provinces. Southern China includes Shanghai, Shandong, Jiangsu, Anhui, Jiangxi, Zhejiang, Fujian, Hubei, Hunan, Guangdong, Guangxi, Hainan, Sichuan, Guizhou, Yunnan Tibet, and Chongqing.

The 19 publications included in this study had a total sample size of 127,660 cases, of which 7,470 were positive cases. The pooled prevalence of AD in children aged 1–7 years in China was 8% (95% CI, 6–9) (Figure 2). Among the 19 studies, there were 9 studies from 1999 to 2012, and the estimated prevalence was 6% (95% CI, 4–8). There were 10 studies between 2013 and 2023, with an estimated prevalence of 10% (95% CI, 7–13). The results showed that the total prevalence in children aged 1–7 years in China during 2013–2023 was higher than that before 2013 (Table 2). In addition, samples were collected from 17 of China's 31 provinces, as other provinces lacked valuable or available epidemiological data on AD prevalence. At the provincial level, the results showed an estimated prevalence of 20% in Zhejiang, Shanxi, and Anhui, which was the highest among the 17 provinces. The remaining 14 provinces, from high to low, followed by Hunan (14%), Hubei (12%), Guangdong (12%), Chongqing (10%), Shanghai (9%), Xinjiang (7%), Liaoning (7%), Beijing (6%), Jiangsu (5%), Hainan (5%), Shanxi (4%), Tianjin (2%), Yunnan (1%), and Sichuan (1%). These provinces were further divided into southern and northern China based on the geographical definition of China along the Qinling–Huai River line. In terms of geographical distribution, the prevalence rates in southern and northern China were 9% and 6%, respectively (Table 2, Figure 3).

**Table 2:** Prevalence of AD and subgroups.

		**No. studies**	**No. positive**	**No. examined**	**% (95% CI)**	**Heterogeneity**		
						χ^2^	***P*** **-value**	**I** ^2^
Sex	Male	16	3,684	59,112	8 (6, 10)	1,472.8	< 0.00001	99%
	Female	16	3,355	57,989	8 (6, 10)	1,454.8	< 0.00001	99%
Sampling time	1999–2012	9	3,980	85,133	6 (4, 8)	1,499.8	< 0.00001	99%
	2013–2023	10	3,490	42,527	10 (7, 13)	1,493.4	< 0.00001	99%
Geographical distribution	Southern China	16	5,804	88,697	9 (8, 11)	3,015.8	< 0.00001	99%
	Northern China	5	1,578	37,468	6 (5, 8)	496.8	< 0.00001	98%
Region	Urban	6	1,083	19,098	6 (3, 8)	199.9	< 0.00001	97%
	Rural	6	175	6,013	4 (2, 6)	124.5	< 0.00001	96%
Exposed to passive smoking	Exposed to passive smoking	5	766	7,042	11 (8, 15)	70.8	< 0.00001	94%
	Not exposed to	5	408	5,825	9 (5, 12)	69.9	< 0.00001	94%
Pet ownership	Exposed to pet	4	123	1,699	11 (4, 18)	135.1	< 0.00001	98%
	Not exposed to	4	452	11,918	5 (3, 8)	58.1	< 0.00001	95%
Plush toy	Exposed to Plush toys	3	237	3,486	9 (5, 14)	40.2	< 0.00001	95%
	Not exposed to	3	237	3,486	6 (3, 9)	48.4	< 0.00001	96%
Feeding pattern	Exclusive breast-feeding	3	295	7,808	4 (1, 9)	0.9	0.62	0%
	Powdered milk	3	87	2,328	7 (2, 12)	26.9	< 0.00001	93%
Family history of disease	Yes	5	418	1,660	20 (9, 31)	108.2	< 0.00001	96%
	No	5	707	9,880	10 (6, 14)	300.1	< 0.00001	99%

### 3.3 Analysis of risk factors for AD in Chinese children

As shown in Table 2, the prevalence of AD was 8% (95% CI, 6–10) in children of both sexes. The prevalence of AD was 6% (95% CI, 3–8) in children from urban areas and 4% (95% CI, 2–6) in children from rural areas; 7% (95% CI, 2–12) in artificially fed children and 4% (95% CI, 1–9) in breastfed children; 20% (95% CI, 9–31) in children with family history of allergies and 10% (95% CI, 6–14) in children without a family history of allergies; 11% (95% CI, 8–15) in children exposed to passive smoking and 9% (95% CI, 5–12) in children not exposed to passive smoking; 11% (95% CI, 4–18) in children from households with pets and 5% (95% CI, 3–8) in children from households without pets; 9% (95% CI, 5–14) in children with plush toys and 6% (95% CI, 3–9) in children without plush toys.

## 4 Discussion

The prevalence of AD is gradually increasing, particularly in the developed countries (35). This study found that the prevalence of AD in Chinese children also showed an increasing trend from 2013 to 2023, which further indicates the growing trend of AD prevalence, and the growth of AD prevalence also indicates its potential harm to Chinese children. The prevalence of AD in children varies worldwide, with Africa, Europe, and Latin America having AD prevalence rates of more than 15% and some countries even exceeding 20% (9–11). In East Asia, studies have shown that the prevalence of AD among preschool children is 9.2%, and the prevalence of AD among children in mainland Japan is 12–13% (36, 37). This study reviewed the pooled prevalence of AD in China from 1999 to 2022 and found that the prevalence of AD among children in China was 8%, which is lower than that in Africa, Europe, Latin America and East Asia. This may be related to the use of diagnostic methods because some children with mild AD may be missed when the Williams diagnostic criteria are used, resulting in a low prevalence (38). The Williams diagnostic criteria were adopted in the included studies, which may have caused some children with mild AD to miss the diagnosis when using the diagnostic criteria, resulting in a low prevalence rate.

This study found that the prevalence of AD in children living in cities was higher than that in children living in rural areas. Air pollution caused by urban industrialization has significantly increased the types and concentrations of allergens to which people are exposed, which may explain the high prevalence of AD. The prevalence of AD in Japanese children has been reported to increase from 15% in 1985 to 24.1% in 1993, indicating that urban industrialization increases the risk of AD in children (39). Studies have found that Australian children living on farms have lower rates of allergic diseases (40). In addition, the urban living environment is dense, with a large number of house decorations and excessive use of air conditioning and carpets, resulting in an increase in the concentration of dust mites, and children are weak in the external environment; therefore, there will be a higher prevalence of AD in urban children (16). In Africa, the prevalence of AD among children living in cities is 1.2%, and that among children living in rural areas is 0.3% (41). These findings suggest that urban air pollution, dietary habits, and exposure to infectious agents are associated with a higher prevalence of AD in urban children. During this decade, with China's rapid industrial development, people's living standards continue to improve. Parents create a better living environment for their children, raise pets, purchase various toys, and expose children to more allergens, which may be the reason for the increase in the prevalence of AD in children from 2013 to 2023 observed in this study.

Plush toys easily absorb pathogenic particles and dust, and the humid environment in the Chaoshan area easily absorbs pathogenic particles and dust, resulting in allergic reactions in children (42). This study found that children with plush toys had a higher prevalence of AD, suggesting that plush toys are a potential trigger for AD development. Studies have found that genetic factors play a role in the development of AD and that if one parent has the disease, their children are at an increased risk (7). This study found that the prevalence of AD increased in children with a family history of the disease, suggesting that genetic factors contribute to the development of AD. It was found that filaggrin (FLG), which encodes intermediate filaggrin associated protein, is a genetic susceptibility gene for AD. Mutations in the FLG gene can lead to dry skin and increased sensitivity. However, individuals who carry the mutated FLG gene will not necessarily develop AD, just have a higher risk of developing AD than those who do not have the mutation (7). Therefore, genetic factors are not the only factors that determine the occurrence of AD, and its pathogenic mechanism needs to be further explored in the future. Passive smoking is a risk factor for AD development, and because tobacco combustion products are common indoor pollutants, they can stimulate allergic reactions in children (43). Studies have shown that nicotine, tar, and other substances in tobacco can increase IgE levels in children and that the body is highly sensitized, increasing the possibility of AD (44). The present study found that children with a history of passive smoking had a higher prevalence of AD, suggesting that children who smoke passively have an increased risk of developing AD. Exclusive breastfeeding reduces the risk of developing AD (45). In this study, we found that the prevalence of AD in Chinese children who were exclusively breastfed was lower than in children who were artificially fed, suggesting that exclusive breastfeeding can reduce the incidence of AD. In addition, this study found that the prevalence of AD in children in pet families was higher than that in non-pet families, which may be because pet fur is very easy to carry pathogenic bacteria and parasites, which can irritate the skin of children, thereby increasing the risk of AD.

According to the results of this study, the prevalence of AD among children in Southern China was higher than that in Northern China. South China has a subtropical monsoon climate, a long rainy season, precipitation, high air humidity, humid environment, and easy breeding of dust mites and mold. In addition, the difference in dietary habits between the north and south of China may also contribute to the difference in the prevalence of AD. People in southern China eat rice as a staple food and eat more fish and shrimp, all of which can be allergenic in some individuals. In this study, the provinces with a high prevalence were Zhejiang, Shanxi, and Anhui, all of which were 20%. The prevalence of the disease varies from 1% to 20% across China's provinces. The differences between the different provinces in China may be related to the local diet, lifestyle, climate, and air quality; however, the specific reasons need to be further analyzed.

This study has some limitations. First, several MeSH terms were used in this study, but not all AD-related publications were covered in the selected database; therefore, there may be some publications that were not retrieved. Secondly, the included publications were from only 17 of China's 31 provinces; therefore, data on the geographical distribution of AD in China are limited. Most of the included studies were published in China. Although these publications met the inclusion criteria, we hope that more English-language publications will be available on the epidemiology of AD. Moreover, because the included studies lacked an analysis of the association between disease severity and risk factors, we do not yet know whether risk factors change with disease severity. Furthermore, most included studies did not have detailed age distribution statistics for the target population; therefore, the prevalence of AD in different age distributions could not be obtained.

## 5 Conclusion

This study investigated the prevalence and epidemiological characteristics of AD in children aged 1–7 years in China and showed that the prevalence of AD in children was 8%, and that the prevalence trend increased over the past 10 years. To the best of our knowledge, this is the first epidemiological analysis of children aged 1–7 years in China. This study enriches the epidemiological data of children with AD in China and worldwide and provides an epidemiological basis for the prevention of AD in children aged 1–7 years.

## Data availability statement

The original contributions presented in the study are included in the article/supplementary material, further inquiries can be directed to the corresponding author.

## Author contributions

XM: Data curation, Methodology, Writing – original draft, Writing – review & editing. ZX: Data curation, Methodology, Writing – original draft, Writing – review & editing. YZ: Funding acquisition, Supervision, Writing – original draft. HS: Methodology, Writing – original draft, Writing – review & editing.
